# Multiparametric MRI for Assessing the Tumor Microenvironment in Head and Neck Cancer: A Narrative Review

**DOI:** 10.3390/medicina62061089

**Published:** 2026-06-04

**Authors:** Csaba Csutak, Călin Schiau, Cristian Dinu, Sebastian Stoia, Georgeta Mihaela Rusu, Lavinia Manuela Lenghel, Delia Doris Donci, Marcela Iojiban

**Affiliations:** 1 Department of Radiology and Imaging, “Iuliu Hațieganu” University of Medicine and Pharmacy, 400012 Cluj-Napoca, Romania; csutakcsaba@yahoo.com (C.C.); mihageorgeta@yahoo.com (G.M.R.); lenghel.manuela@gmail.com (L.M.L.); delia.muntean@umfcluj.ro (D.D.D.); 2 Department of Radiology and Medical Imaging, Cluj County Emergency Clinical Hospital, 400347 Cluj-Napoca, Romania; 3 Department of Maxillofacial Surgery and Implantology, “Iuliu Hațieganu” University of Medicine and Pharmacy, 400347 Cluj-Napoca, Romania; cristian.dinu@umfcluj.ro (C.D.); sebastian.stoia@umfcluj.ro (S.S.); 4 Department of Radiology and Imaging. Nuclear Medicine, “Iuliu Hațieganu” University of Medicine and Pharmacy, 400162 Cluj-Napoca, Romania; mmarcela1612@gmail.com

**Keywords:** head and neck cancer, dynamic contrast-enhanced MRI, arterial spin labeling, diffusion-weighted imaging, intravoxel incoherent motion, diffusion kurtosis imaging, amide proton transfer imaging, blood oxygen level-dependent MRI, oxygen-enhanced MRI, tumor hypoxia

## Abstract

*Background and Objectives:* Head and neck cancers are heterogeneous malignancies with variable biological behavior and treatment response, contributing to high morbidity and mortality. Conventional imaging techniques are limited in their ability to capture tumor biology, highlighting the need for advanced functional imaging. This review aims to evaluate the role of multiparametric magnetic resonance imaging (MRI) in characterizing the tumor microenvironment. *Materials and Methods:* A narrative review was conducted based on a targeted literature search of databases, including PubMed and Google Scholar. Studies addressing advanced MRI techniques for assessing tumor cellularity, vascularity, molecular features, and oxygenation were selected and analyzed. *Results:* Perfusion techniques, such as dynamic contrast-enhanced MRI (DCE-MRI) and arterial spin labeling (ASL), provide a quantitative assessment of tumor vascularity and show value in predicting treatment response. Diffusion-based methods, including diffusion-weighted imaging (DWI), intravoxel incoherent motion (IVIM), and diffusion kurtosis imaging (DKI), enable evaluation of tissue cellularity and heterogeneity. Molecular approaches, such as chemical exchange saturation transfer (CEST) and amide proton transfer (APT), offer insights into protein content and proliferation. Oxygenation-sensitive techniques, such as blood oxygenation level dependent MRI (BOLD MRI) and oxygen-enhanced MRI (OE-MRI), allow non-invasive assessment of tumor hypoxia. *Conclusions:* Multiparametric MRI provides a comprehensive and biologically relevant evaluation of the tumor microenvironment in head and neck cancer, with potential to improve treatment prediction and support personalized therapeutic strategies.

## 1. Introduction

Head and neck cancers are a significant global health issue, ranking among the most prevalent malignancies worldwide and accounting for hundreds of thousands of new cases reported each year [1,2]. These cancers arise from diverse anatomical sites, including the lip, oral cavity, nasopharynx, oropharynx, hypopharynx, and larynx [1,2]. Major risk factors for tumor development include tobacco smoking, alcohol consumption, and oncogenic viral infections such as human papillomavirus (HPV) and Epstein–Barr virus (EBV), which contribute to their pathogenesis [2]. Despite advancements in surgical and oncological therapies, morbidity and mortality rates remain high, largely due to the substantial biological and clinical heterogeneity of these tumors [3].

Imaging plays a central role in the diagnosis, staging, and management of head and neck cancer [4]. Imaging techniques, such as computed tomography (CT) and magnetic resonance imaging (MRI), complement each other, providing information that can influence clinical decision-making [4]. MRI, in particular, offers superior soft-tissue contrast and facilitates the evaluation of both anatomical and functional tumor characteristics, such as tissue cellularity and vascularity [4]. However, challenges remain, including selecting optimal imaging modalities, standardizing acquisition protocols, clarifying the role of advanced functional MRI, and developing quantitative post-treatment surveillance strategies [4].

Conventional imaging techniques primarily provide anatomical information and often fail to capture the underlying tumor biology. Head and neck cancers exhibit significant intratumoral heterogeneity, with distinct subtypes characterized by variable cellularity, perfusion, and oxygenation. These variations can influence the treatment response [5]. Notably, tumor hypoxia is recognized as a critical factor associated with resistance to radiotherapy and poor clinical outcomes [6].

Regarding this new approach to tumor behavior, there is increasing interest in advanced MRI techniques capable of non-invasively characterizing the tumor microenvironment. Quantitative MRI parameters, including diffusion, perfusion, and oxygenation-related metrics, have shown potential as surrogate biomarkers of tissue cellularity, vascularity, and hypoxia [5,7]. Moreover, integrating multiparametric imaging approaches may improve tumor characterization and enable more accurate prediction of treatment response and clinical outcomes [7].

This review aims to provide a thorough overview of advanced MRI techniques for characterizing the tumor microenvironment in head and neck cancers, and to discuss their potential role as non-invasive imaging biomarkers for predicting treatment response and guiding personalized therapeutic strategies. Unlike previous reviews that primarily focused on individual MRI techniques, this review offers an integrative overview of multiparametric MRI biomarkers within the broader context of tumor microenvironment characterization and clinical translation in head and neck cancer.

## 2. Materials and Methods

This study was conducted as a narrative review, guided by a targeted literature search performed from August 2025 to February 2026. Articles pertaining to advanced MRI techniques for assessing the tumor microenvironment in head and neck cancer were retrieved from databases such as PubMed and Google Scholar, covering the period from January 2010 through February 2026.

To guide the search, terms such as “head and neck cancer”, “MRI”, “diffusion-weighted imaging”, “intravoxel incoherent motion”, “diffusion kurtosis imaging”, “dynamic contrast-enhanced MRI”, “arterial spin labeling”, “chemical exchange saturation transfer”, “amide proton transfer”, “blood oxygen level-dependent MRI”, and “oxygen-enhanced MRI” were utilized.

The research was restricted to articles written in English. The literature was examined in accordance with the purpose of this review, and the most relevant and informative articles focusing on MRI-derived biomarkers, with emphasis on tumor cellularity, perfusion, molecular features, and oxygenation, were included. While priority was given to head and neck cancers, selected studies on other tumor types and one case report were also included to provide relevant methodological insights.

**Figure 1:**
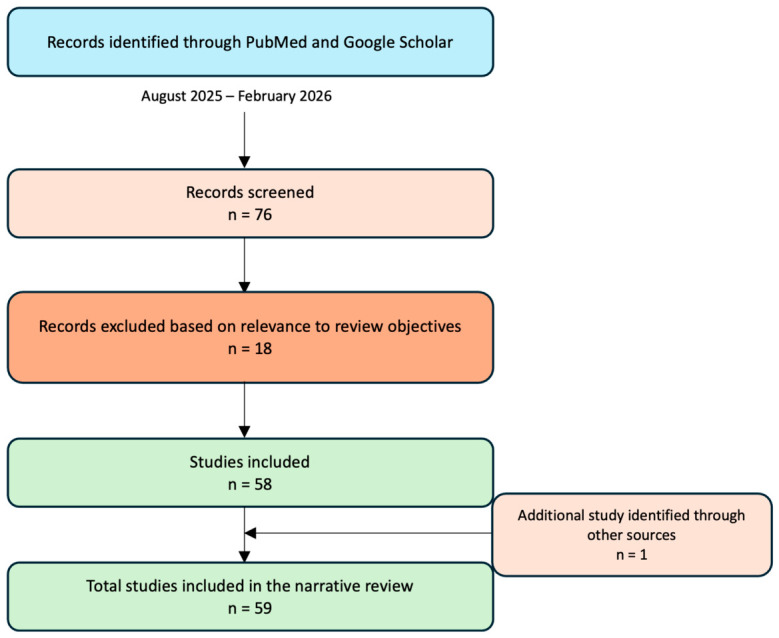
Flow diagram of the literature search and study selection process for the narrative review.

A study selection diagram is provided in Figure 1.

## 3. Perfusion MRI Techniques

Perfusion MRI techniques offer important functional information on tumor vascularity and microcirculation in head and neck cancer, with dynamic contrast-enhanced MRI (DCE-MRI) and arterial spin labeling (ASL) being the principal methods for quantitative perfusion assessment.

### 3.1. DCE-MRI

Dynamic contrast-enhanced magnetic resonance imaging (DCE-MRI) enables quantitative evaluation of tumor perfusion, vascular permeability, and the extravascular extracellular space (EES). This is achieved using specific parameters: Ktrans quantifies the volume transfer constant from plasma to the EES, indicating the rate of contrast agent movement into tissue; Kep measures the rate at which contrast returns from the EES to plasma, reflecting washout dynamics; and Ve estimates the volume fraction of the EES, providing insight into the relative space outside cells [8,9,10]. The acquisition time of the DCE-MRI sequence varies across different protocols, ranging from 4 min and 26 s to 6 min and 10 s [8,11,12].

**Figure 2:**
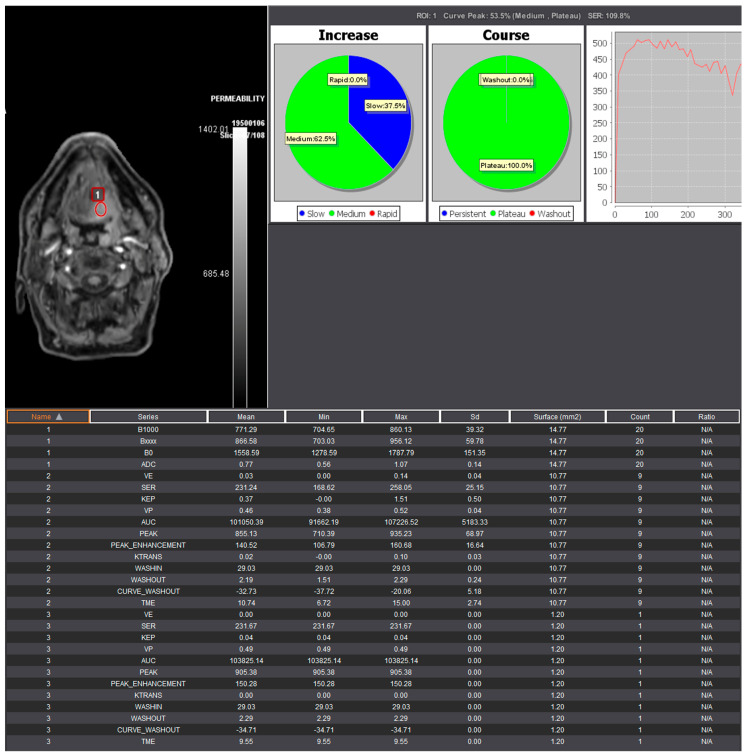
Patient with HPV-negative squamous cell carcinoma of the oropharynx and oral cavity; low Ktrans value indicating a poor response to oncological treatment. The red circle indicates the region of interest (ROI) placed within the tumor.

Building on this, DCE-MRI parameters can predict treatment response by reflecting tumor physiology. Higher Ktrans values, indicating increased transfer of contrast from plasma to tissue, and elevated vascular endothelial growth factor (VEGF) levels, both measured at baseline and early in treatment, allow distinction of responders from non-responders in HPV-negative oropharyngeal squamous cell carcinoma, using cut-off values of 0.259 min^−1^ for Ktrans and 62.5 pg/mL for VEGF (Figure 2) [11].

Furthermore, DCE-MRI also provides semiquantitative parameters, each with specific prognostic significance. The area under the contrast concentration–time curve at 60 s (AUC60p95) represents the 95th percentile of contrast uptake within the first 60 s, quantifying the most avid enhancement, and has prognostic value. Lower pre-treatment AUC60p95 values, reflecting poor early perfusion, are associated with reduced overall survival, linking this metric with poor treatment response [13].

**Figure 3:**
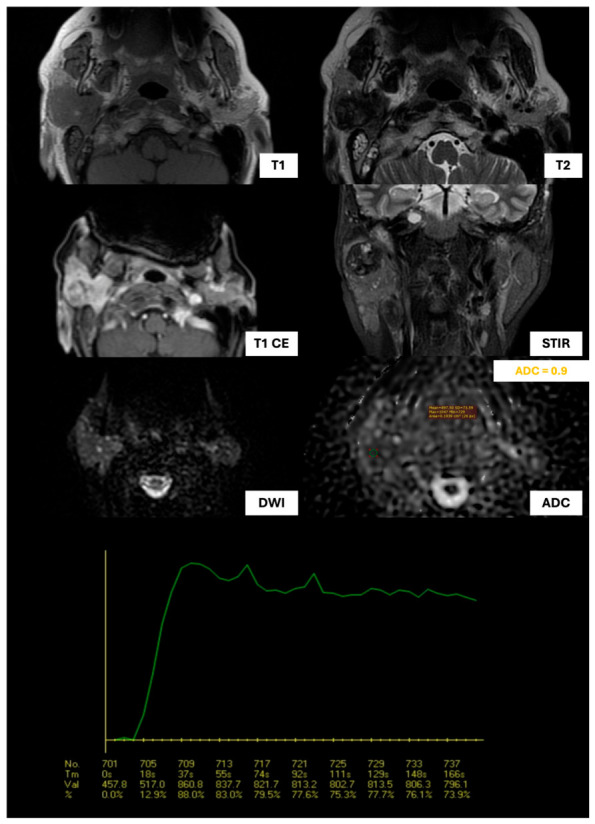
Salivary duct carcinoma of the right parotid gland, demonstrating a type C enhancement curve on DCE-MRI. The green circle indicates the region of interest (ROI) placed within the tumor in order to measure the ADC value.

**Figure 4:**
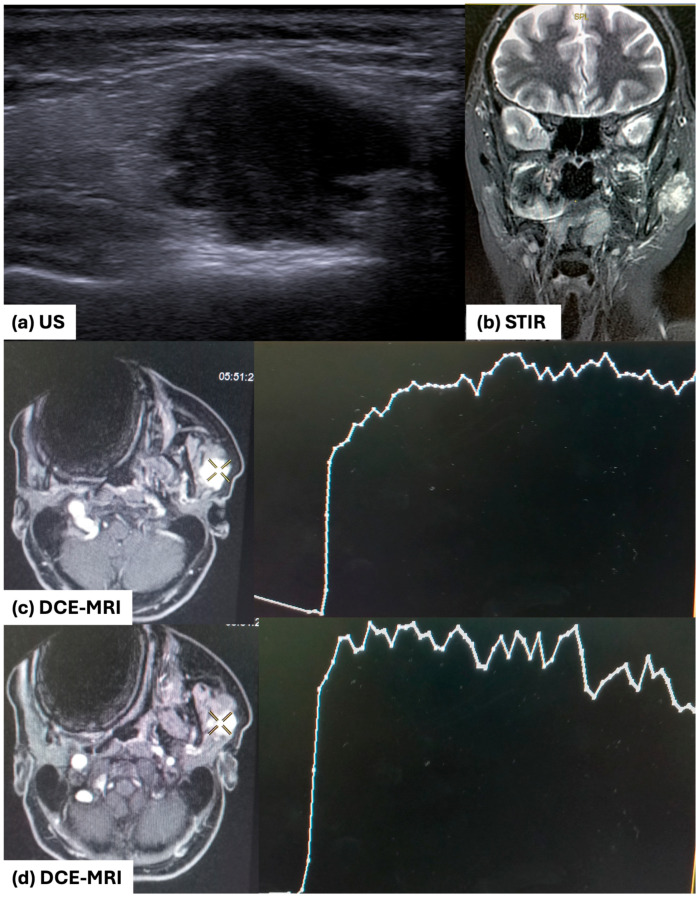
A 40-year-old patient with a slowly enlarging left parotid mass over the past five years—(**a**) Ultrasound (US) demonstrates an inhomogeneous lesion with irregular margins; (**b**) STIR MRI confirms the heterogeneous appearance. DCE-MRI shows two types of enhancement curves: (**c**) type A and (**d**) type C. Regions of interest were placed across different areas of the tumor, reflecting its intratumoral heterogeneity. Fine-needle aspiration suggested a benign tumor; however, following surgical excision, the final histopathological diagnosis was mucoepidermoid carcinoma.

Dynamic contrast-enhanced MRI enables evaluation of tumor enhancement patterns and contributes to differential diagnosis between benign and malignant lesions. There are four types of enhancement patterns described in the literature, based on time to reach peak enhancement intensity (TTP, time-to-peak) and washout ratio [14]. Type A is characterized by TTP > 120 s, corresponding to a gradual enhancement, associated with a washout < 30%, suggestive of a benign nature, characteristic of pleomorphic adenoma [14,15]. Type B presents with TTP ≤ 120 s and washout ≥ 30% and is frequently encountered in Warthin tumors. Type C is defined by TTP ≤ 120 s and washout < 30%, a characteristic feature of malignant tumors (Figure 3 and Figure 4) [14,15]. Finally, type D corresponds to the absence of enhancement and is associated with cystic lesions [14].

In addition to assessing tumor response, DCE-MRI is sensitive to tissue changes following radiotherapy by leveraging specific parameters. The mean Ve, which estimates the fraction of the EES, differs significantly between mandibular regions exposed to high versus low radiation doses (*p* = 0.00013). Higher Ve values in areas receiving more than 60 Gy indicate expansion of the extracellular space due to radiation-induced tissue injury, helping to enable early detection of mandibular osteoradionecrosis [16].

### 3.2. ASL

Arterial spin labeling (ASL) is a non-invasive MRI perfusion technique. It uses magnetically labeled arterial blood water as an endogenous tracer, allowing repeated measurement of intratumoral blood flow without administering contrast agents. ASL is most commonly used for cerebral perfusion [17]. The acquisition times differ among protocols, ranging from 1 min and 31 s to 5 min [17,18,19]. 

This technique has demonstrated utility in predicting treatment response in head and neck cancers. Specifically, in nasopharyngeal carcinoma, higher pre-treatment tumor blood flow (pre-TBF) and significant changes during treatment (ΔTBF) are significantly associated with treatment response (*p* < 0.01). Furthermore, pre-TBF shows good predictive performance in predicting sensitivity to chemoradiotherapy and residual tumor status (AUCs of 0.845 and 0.831, respectively) [17].

ASL-derived parameters correlate with those obtained from DCE-MRI. Tumor blood flow measured using multidelay ASL shows a significant positive correlation with DCE-MRI parameters (Ktrans, Kep) (r_s_ = 0.61–0.71, *p* < 0.001), supporting ASL as a surrogate biomarker of tumor vascularity without the need for contrast administration [12].

In the post-treatment setting, particularly in brain metastases, ASL can help differentiate viable residual tumor from treatment-related changes. Tumors demonstrate higher perfusion than post-radiotherapy necrosis (*p* < 0.0001), with good diagnostic performance (AUROC up to 0.90) [20], suggesting a potential future research direction for head and neck cancers.

Additionally, ASL can characterize tumor vascularity and vascular anatomy. Hypervascular tumors, such as paragangliomas, demonstrate increased ASL signal [19], while ASL-based angiography can visualize small arterial branches more effectively than CT angiography [21].

## 4. Advanced Diffusion MRI Techniques

Diffusion MRI techniques, including conventional DWI and derived models such as IVIM and DKI, enable a more comprehensive characterization of tissue microstructure and the tumor microenvironment.

### 4.1. DWI/ADC

Diffusion-weighted magnetic resonance imaging (DWI) is a non-invasive technique that assesses the Brownian motion of water molecules within tissue [22,23,24]. The b-value, a parameter of DWI sequences, quantifies the image’s sensitivity to water-molecule motion. Acquiring DWI images at multiple b-values enables the calculation of apparent diffusion coefficient (ADC) maps [23]. This approach is valuable for tissue characterization, as each structure has distinct water mobility based on its architectural properties [23,24]. ADC values vary with tissue composition and microstructural features. Tissues with high cellularity and reduced extracellular space generally have lower ADC values, indicating restricted diffusion [23]. Hypercellular tumor lesions are thus characterized by restricted water diffusion [23]. Image interpretation may be conducted qualitatively, by evaluating signal intensity on DWI and ADC maps, or quantitatively, by measuring ADC values [25]. The total scan time is approximately 2 min [22].

**Figure 5:**
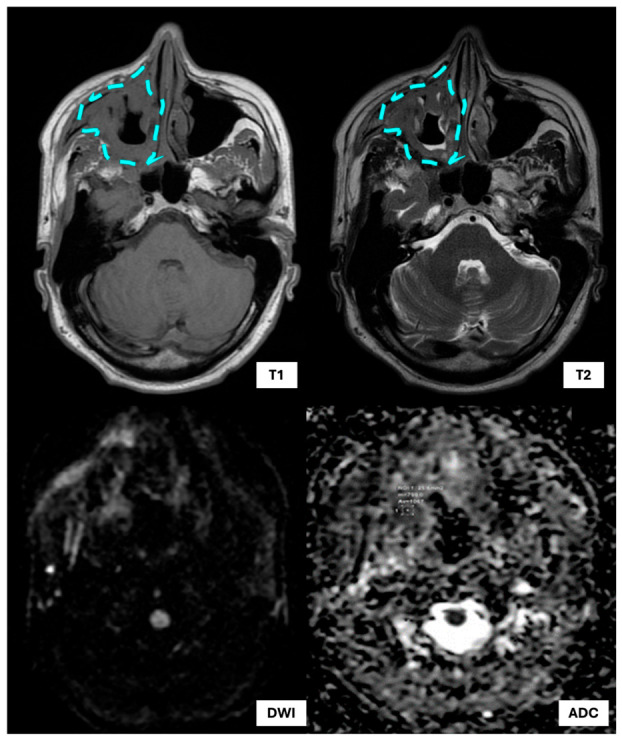
A 39-year-old patient presenting with right cheek anesthesia. MRI demonstrates a circumferential soft tissue mass with low signal intensity on T1- and T2-weighted images, involving the right maxillary sinus (blue dashed line) and extending into adjacent structures, including the ethmoidal cells, nasal cavity, pterygopalatine fossa, skull base, and sphenoidal sinus. DWI and the corresponding ADC map show restricted diffusion (ADC value = 1.06 × 10^−3^ mm^2^/s). The final diagnosis was adenoid cystic carcinoma.

**Figure 6:**
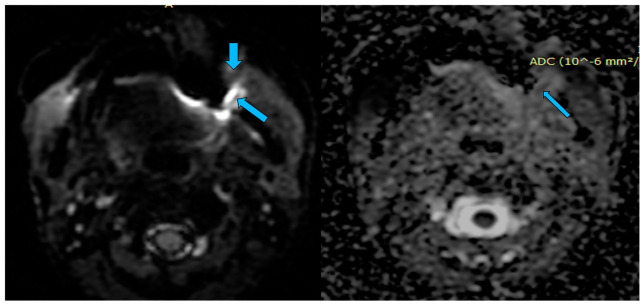
A 60-year-old patient with recurrent squamous cell carcinoma. Diffusion-weighted imaging demonstrates diffusion restriction, consistent with tumor recurrence (blue arrows).

No consensus has been established on the ADC cut-off value for differentiating benign from malignant tumors in the head and neck region, with reported threshold values ranging from approximately 0.7 × 10^−3^ mm^2^/s to 1.5 × 10^−3^ mm^2^/s [26,27,28,29,30]. Malignant tumors generally demonstrate lower ADC values. ADC holds a crucial role not only in diagnosis (Figure 5), but also in prognostic assessment, prediction of treatment response, detection of tumor recurrence (Figure 6), and differentiation of tumor recurrence from post-treatment changes [24,25,26,27,28,29,30,31].

Conventional DWI sequences rely on mono-exponential modeling of water diffusion. This approach provides limited information in complex biological tissues, where diffusion behavior may deviate from a Gaussian distribution and be influenced by microvascular perfusion [32,33]. To address these limitations, advanced diffusion models such as intravoxel incoherent motion (IVIM) and diffusion kurtosis imaging (DKI) have been developed, thereby facilitating improved characterization of the tumor microenvironment [34,35,36].

### 4.2. IVIM

**Figure 7:**
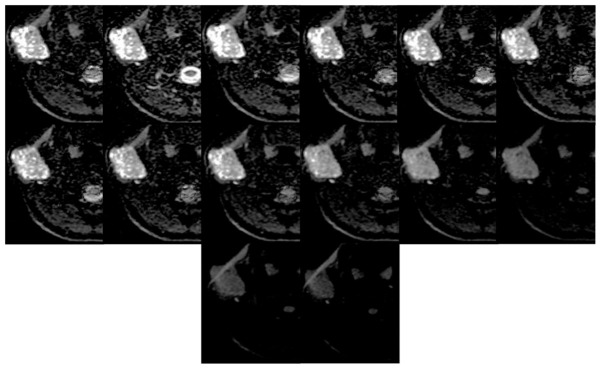
IVIM diffusion-weighted imaging acquired using 14 b-values illustrating progressive signal attenuation.

Among these techniques, intravoxel incoherent motion (IVIM) is an advanced diffusion-weighted MRI method that uses multiple b-values and models signal decay according to a bi-exponential function (Figure 7). This separates true molecular diffusion from perfusion-related pseudo-diffusion. IVIM provides three quantitative parameters: the true diffusion coefficient (D), which reflects tissue diffusivity and cellularity; the pseudo-diffusion coefficient (D*), related to microvascular blood flow; and the perfusion fraction (f), representing the fractional volume of microvascular perfusion within a voxel [35,37]. This separation enables simultaneous characterization of tissue cellularity and tumor vascularity. As a result, it contributes to the assessment of tumor heterogeneity [35,37]. Total scan durations differ among protocols, ranging from 2 min and 55 s to 8 min [35,38,39]. 

The clinical utility of IVIM has been explored in several studies. IVIM may improve diagnostic performance in local tumor staging. In nasopharyngeal carcinoma, combining IVIM-DWI with conventional MRI improves the detection of skull-base invasion. The combination achieves an AUC of 0.947, with a sensitivity of 92.6%, and a specificity of 96.8% [38].

IVIM also shows potential for predicting treatment response. In a meta-analysis including 347 patients, both pre-treatment D values and changes in D after treatment demonstrated good diagnostic performance for predicting early response to chemoradiotherapy, with a sensitivity of 76% and specificity of 81% for pre-treatment D, and a sensitivity of 70% and specificity of 82% for changes in D [39]. Increases in D and ADC values during treatment have been associated with a favorable response, reflecting reduced tumor cellularity due to apoptosis [40,41]. Early changes in diffusion parameters during treatment may allow identification of patients with complete versus incomplete response, enabling treatment adaptation [41]. Perfusion-related parameters, such as the perfusion fraction (f), showed greater variability, suggesting that D is a more robust predictor of treatment response [39].

Fujima et al. demonstrated correlations between IVIM-derived perfusion parameters and parameters obtained from dynamic contrast-enhanced MRI, such as f and tumor blood volume, as well as f × D* and tumor blood flow in head and neck squamous cell carcinoma [35]. Jia et al. observed the highest correlations between f and enhancement amplitude (r_s_ = 0.633, *p* < 0.001) and between f and maximum slope of increase (r_s_ = 0.598, *p* = 0.001) [42]. These observations suggest that IVIM may serve not only as a marker of tumor cellularity but also as a surrogate imaging biomarker for perfusion assessment without contrast administration [35]. However, these correlations are not consistently reported in the literature, as perfusion-related parameters are highly sensitive to acquisition parameters and fitting methods [34].

### 4.3. DKI

Diffusion kurtosis imaging (DKI) is an advanced diffusion-weighted MRI technique derived from diffusion models that extends the mono-exponential approach of conventional DWI by accounting for the non-Gaussian behavior of water diffusion in complex biological tissues. It provides quantitative parameters including diffusivity (Dapp), which reflects water diffusion corrected for non-Gaussian effects, and kurtosis (Kapp), which quantifies the deviation from Gaussian diffusion and serves as a marker of tissue microstructural complexity and tumor heterogeneity [36,43,44]. Depending on the model used, these parameters may also be expressed as directional metrics, such as Dmean, Daxis, Drad, and Kmean, Kaxis, and Krad [36]. Reported acquisition times range from 8 min and 21 s to 8 min and 53 s [36,43].

DKI has shown utility in differentiating benign from malignant salivary gland tumors. Diffusivity values are significantly lower in malignant tumors than in pleomorphic adenomas, and a cut-off of 1.46 × 10^−3^ mm^2^/s achieves an AUC of 0.96 and an accuracy of 92%. In addition, kurtosis values may help differentiate Warthin tumors from pleomorphic adenomas and other benign salivary gland tumors [44].

DKI may also help predict response to radiotherapy, though reported findings vary with timing and the clinical endpoint evaluated. In a study of nasopharyngeal carcinoma patients, pre-treatment diffusivity values were significantly higher and kurtosis significantly lower in responders compared with non-responders when assessing early response. Among evaluated parameters, Krad had the best predictive performance, with a cut-off of 0.76, sensitivity of 71.4%, specificity of 93.7%, and an AUC of 0.897 [36]. Studies assessing long-term outcomes showed that advanced nasopharyngeal carcinoma with lower diffusivity and higher kurtosis—indicating increased cellularity and heterogeneity—was linked to improved progression-free survival [45]. These findings are not necessarily contradictory. They reflect different aspects of tumor biology, including short-term radiosensitivity and long-term disease behavior. Differences in clinical endpoints, assessment timing, and patient cohorts also influence outcomes [36,45].

DKI helps further characterize tissue by providing information beyond what diffusion alone can provide. Diffusivity (Dapp) correlates negatively with Kep (the volume transfer constant between the extravascular extracellular space and the blood plasma) (r_s_ = −0.510) and positively with Ve (the extravascular extracellular volume fraction) (r_s_ = 0.418) [43]. This indicates that increased diffusion restriction is associated with higher vascular permeability and a reduced extracellular space. Kurtosis (Kapp) correlates positively with entropy-based perfusion parameters (r_s_ = 0.407), suggesting that increased microstructural complexity is associated with more heterogeneous, disorganized perfusion patterns [43].

These findings show the potential of DKI as a tumor biomarker, capturing both cellular density and microstructural heterogeneity.

## 5. Molecular MRI Techniques

Chemical exchange saturation transfer (CEST) MRI is a molecular imaging technique. It enables indirect detection of endogenous metabolites containing exchangeable protons, such as amide groups in proteins and peptides. This is achieved through their selective saturation and subsequent proton exchange with water [46]. This mechanism allows amplification of signals from endogenous proteins and peptides that are not directly visible on conventional MRI. In the oncologic setting, CEST MRI provides information on tumor microenvironment features, including protein content and pH-related processes [47].

Amide proton transfer (APT) imaging is the most widely used CEST application. It reflects mobile proteins and peptides with a resonance frequency offset of approximately 3.5 parts per million (ppm) relative to water, corresponding to the amide proton chemical shift. APT signal intensity is expressed as a percentage of magnetization transfer asymmetry (MTRasym). This reflects the relative contribution of amide proton exchange to the bulk water signal and serves as a biomarker of tumor cellularity and proliferation [48]. Reported acquisition times in brain imaging are 7–8 min [48].

In head and neck cancers, APT has been shown to be useful in differentiating tumor tissue from normal structures. Tumors demonstrate increased APT signal intensity [49].

CEST-derived parameters have shown potential in predicting treatment response. In nasopharyngeal carcinoma, MTR values obtained from 3D CEST imaging were significantly higher in patients with residual disease compared to responders after chemoradiotherapy (34.74 ± 1.54% vs. 31.24 ± 5.21%, *p* = 0.003). These values also demonstrated good predictive performance (AUC = 0.818) [50].

Beyond head and neck tumors, APT imaging has also been shown to reflect tumor aggressiveness and proliferation in other malignancies. Higher values are observed in malignant compared to benign tumors (4.62 ± 1.43% vs. 2.26 ± 1.07%, *p* < 0.001) [51]. In addition, APT values are positively correlated with Ki-67 expression (r_s_ = 0.546, *p* = 0.002), suggesting its role as a non-invasive biomarker of tumor proliferation [51] and a promising direction for further research in head and neck cancers.

Overall, CEST/APT MRI provides a non-invasive approach for assessing the molecular tumor microenvironment, complementing diffusion and perfusion imaging by offering insights into protein content and cellular proliferation, although technical limitations, such as magnetic field inhomogeneity and the lack of standardized acquisition protocols, remain significant challenges [46].

## 6. Oxygenation MRI Techniques

Tumor hypoxia is an imbalance between oxygen supply and demand. It is a major cause of treatment resistance in head and neck cancers, as it reduces tumor radiosensitivity [52,53]. Blood oxygen level-dependent (BOLD) MRI and oxygen-enhanced (OE) MRI can assess tumor oxygenation without invasive procedures. The reported scan duration for OE-MRI is less than 10 min [6].

BOLD MRI evaluates tissue oxygenation indirectly. It measures the paramagnetic effect of deoxyhemoglobin, which changes the transverse relaxation rate R2* (1/T2*). Higher R2* values usually mean more tissue hypoxia. In contrast, OE-MRI measures changes in the longitudinal relaxation rate R1 (1/T1) during oxygen inhalation. This reflects the amount of dissolved oxygen that reaches tissues [53].

OE-MRI is useful for evaluating head and neck cancer. McCabe et al. reported scan times of less than 10 min, a median ΔT1 of −3.5%, and a median hypoxic fraction of 30.7% in head and neck squamous cell carcinoma. Radiotherapy-resistant tumors had higher hypoxic fractions (59.0%) than radiotherapy-responsive tumors (36.8%) [6].

Furthermore, dynamic assessment during treatment demonstrates OE-MRI’s potential. Hypoxic tumor volume drops from 11.3 cm^3^ before radiotherapy to 5.9 cm^3^ at week 4 (*p* < 0.001) [54].

BOLD MRI has prognostic value. In metastatic lymph nodes that respond to chemoradiotherapy, higher baseline R2* values (a measure of magnetic signal loss related to tissue oxygenation) and a measurable change in R2* during 100% oxygen breathing are linked to treatment response. Conventional imaging parameters, such as lymph node size or shape, are not predictive. This may seem paradoxical, since oxygen inhalation should lower deoxyhemoglobin. However, it likely shows the presence and functionality of tumor blood vessels, not worsening hypoxia. In responsive lymph nodes, a change in R2* means the vascular environment is active. In contrast, the absence of change in R2* in non-responsive lymph nodes indicates poor perfusion and ongoing hypoxia, which may lead to treatment resistance [55].

MRI techniques that assess oxygenation give complementary information about tumor hypoxia. OE-MRI shows oxygen delivery to tissues more directly. BOLD MRI gives information about deoxyhemoglobin and tumor vascular characteristics. However, these methods need more validation and standardization before they can be used in routine clinical care [52,53].

A summary of each MRI technique utilized for evaluating the tumor microenvironment in head and neck cancer is provided in Table 1.

## 7. Current Limitations

Despite increasing evidence supporting the clinical utility of multiparametric MRI in head and neck cancer, several significant limitations continue to hinder the adoption of advanced quantitative imaging biomarkers. A primary challenge is the variability in scanner platforms, acquisition protocols, and post-processing methods [4,43,56]. While diffusion-weighted imaging and dynamic contrast-enhanced MRI are commonly employed to characterize head and neck tumors, the use of advanced diffusion and perfusion techniques, such as intravoxel incoherent motion (IVIM), diffusion kurtosis imaging (DKI), arterial spin labeling (ASL), and tumor oxygenation methods, remains limited. These advanced techniques are not universally available across scanners, and standardized acquisition protocols have yet to be established. Additionally, inconsistencies in b-value selection, temporal resolution, and fitting algorithms can substantially affect reproducibility and quantitative reliability, thereby limiting direct comparisons across studies and impeding broader multicenter validation efforts [25,32,57].

Clinical implementation also varies among different MRI techniques. Diffusion-weighted imaging, including ADC analysis, is already widely integrated into routine head and neck MRI because of its established role in lesion characterization, treatment response, and post-treatment surveillance, along with its short acquisition time and broad availability [25]. Dynamic contrast-enhanced MRI is also increasingly used in specialized oncologic imaging centers to evaluate tumor perfusion with respect to contrast administration, vascular permeability, and treatment response prediction [8,35,58]. Nonetheless, broader embracement of quantitative and perfusion biomarkers remains limited by the absence of uniform acquisition, analysis, and reporting recommendations [4,25].

In contrast, quantitative diffusion restriction, arterial spin labeling, diffusion kurtosis imaging, amide proton transfer imaging, and oxygenation-sensitive MRI are inconsistently integrated into MRI protocols within standard clinical practice. Protocol heterogeneity constitutes the primary impediment to routine application.

One reason for variability is the cut-off values. Threshold values for various quantitative parameters, such as ADC values for malignancy [26,27,28,29,30], Ktrans for treatment response prediction [10,11], or IVIM-derived D values [39,42], are reported from single-center study cohorts utilizing specific scanner platforms and protocols. This variability complicates the reliable implementation of findings across different institutions [56]. Variations exist in the b-values and the number of b-values employed across distinct diffusion restriction methodologies [26,27,28,29,30,33,34,35,36,37,42,43].

Analysis of dynamic contrast-enhanced MRI also differs due to the use of various pharmacokinetic models (standard Tofts model, extended Tofts model, and Brix model) [8,10,11,15] or through semiquantitative approaches utilizing time-intensity curve analysis [13,14]. Acquisition parameters must be considered when adopting these techniques, given that scanners have different magnetic field strengths ranging from 1.5 T to 3 T [8,10,11,12,17,18,29,35,49,50], temporal resolution differs across studies [10,11,12,43], and the contrast agents and doses used vary [10,11,12,15].

Another significant impediment is the use of single versus multidelay post-labeling approaches in ASL techniques, which impacts the precision of tumor blood flow quantification [17,18].

Similarly, for CEST and APT imaging, acquisition strategies range from two- (2D) to three-dimensional (3D) protocols, and derived parameters are further affected by magnetic field inhomogeneity [46,49,50].

Oxygenation-sensitive techniques also exhibit variability by measuring different parameters. BOLD imaging focuses on deoxyhemoglobin concentration via R2*, whereas OE-MRI quantifies dissolved oxygen through ΔT1, employing distinct acquisition sequences, even when addressing the same clinical challenge of tumor hypoxia assessment [6,52,53,55].

Furthermore, the timing of the MRI acquisition relative to treatment substantially influences quantitative parameter values and their predictive performance, with no consensus on standardized imaging time points for treatment response assessment in head and neck cancer [39,40,41].

## 8. Future Directions

Although quantitative DWI and DCE-MRI techniques are widely used across institutions, the field remains fragmented largely due to protocol heterogeneity. There is still a need for agreed-upon guidelines for acquiring, analyzing, and reporting findings in a standardized manner so they can become part of routine clinical practice [25]. The formal standardization pathway includes phantom-based acquisition standardization, followed by repeatability and reproducibility validation [56].

Some may contend that the acquisition duration is excessively long in the context of emerging imaging modalities. However, McCabe successfully achieved scan times of less than ten minutes for OE-MRI [6].

Given the rapid progression of imaging technology, the availability of multiparametric MRI and hybrid imaging modalities is increasing within research institutions [52]. The analysis of superimposed data acquired through different modalities in hybrid imaging poses a significant challenge necessitating artificial intelligence-driven techniques aimed at quantifying the intratumoral microenvironment and predicting patient outcomes [52]. While MRI continues to serve as the primary modality in locoregional assessment of head and neck cancer, supplementary imaging techniques may be required depending on the specific clinical question. For example, other imaging modalities, such as computed tomography, ultrasound, and echocardiography, may be of value in the setting of suspected disseminated disease [59]. The integration of MRI with PET-CT could offer additional biological insights into tumor metabolism and treatment response [7].

Finally, to enable global implementation, prospective multicenter validation is essential, given that the majority of existing studies are single-center and retrospective [36,39,45].

A summary of the evidence strength and limitations of MRI techniques utilized in evaluating the tumor microenvironment in head and neck cancer is provided in Table 2.

## 9. Conclusions

Multiparametric MRI offers a comprehensive, non-invasive framework for characterizing the tumor microenvironment in head and neck cancer, overcoming the limitations of conventional anatomical imaging. Among diffusion-based techniques, DWI and ADC analysis are the most clinically mature, with an established role in tumor characterization, assessment of treatment response, and post-treatment surveillance. Advanced diffusion models provide additional diagnostic value. By integrating IVIM with conventional MRI, the combined examination achieves excellent performance in detecting skull-base invasion in nasopharyngeal carcinoma, while DKI demonstrates good performance in predicting early radiotherapy response. Perfusion techniques can evaluate treatment response, with Ktrans and pre-treatment ASL tumor blood flow emerging as promising predictors. At the molecular level, CEST-derived MTR values successfully distinguished residual disease from complete response following chemoradiotherapy, with good performance. Oxygenation-sensitive modalities provide non-invasive evaluation of tumor hypoxia, a recognized contributor to radiotherapy resistance, although these remain in the investigational stage.

Despite this evidence, several challenges continue to limit the wide routine implementation. Variability in protocols across scanner platforms, acquisition parameters, and post-processing methods substantially impede reproducibility. Reported cut-off values for key parameters are derived from single-center cohorts and cannot yet be generalized. Advanced techniques, such as IVIM, DKI, ASL, CEST, BOLD, and OE-MRI, remain inconsistently incorporated into clinical protocols, with standardized acquisition guidelines yet to be established. As a narrative review, this work presents a descriptive synthesis of the available evidence, and a systematic approach was beyond its scope. The findings should be interpreted in this context.

Progress toward clinical translation will necessitate coordinated standardization efforts and prospective multicenter validation of the most promising biomarkers. With ongoing advances in these areas, multiparametric MRI demonstrates potential for characterizing the tumor microenvironment and supporting personalized treatment strategies to enhance outcomes for patients with head and neck cancer.

## Figures and Tables

**Table 1:** MRI techniques utilized for assessing the tumor microenvironment in head and neck cancer.

Technique	Key Parameters	Main Clinical Applications	Biological Correlate
DCE-MRI	Ktrans, Kep, Ve	Treatment response	Perfusion
	TIC	Differential diagnosis	
ASL	TBF	Treatment response	Perfusion
DWI/ADC	Signal intensity	Diagnosis Treatment response Post-treatment surveillance	Diffusion
	ADC value		
IVIM	D, D*, f	Local tumor staging Treatment response	Diffusion and perfusion
DKI	Dapp, Kapp	Differential diagnosis Treatment response	Diffusion
APT/CEST	MTRasym	Differential diagnosis	Molecular level
BOLD-MRI	R2*	Treatment response	Oxygenation
OE-MRI	ΔT1	Treatment response	Oxygenation

DCE-MRI = dynamic contrast enhanced-magnetic resonance imaging; Ktrans = the volume transfer constant from plasma to the extravascular extracellular space; Kep = the rate at which contrast returns from the extravascular extracellular space to plasma (washout dynamics); Ve = the volume fraction of the extravascular extracellular space; TIC = time-intensity curves; ASL = arterial spin labeling; TBF = tumor blood flow; DWI/ADC = diffusion-weighted imaging/apparent diffusion coefficient; IVIM = intravoxel incoherent motion; D = true diffusion coefficient; D* = pseudo-diffusion coefficient; f = perfusion fraction; DKI = diffusion kurtosis imaging; Dapp = diffusivity; Kapp = kurtosis; APT = amide proton transfer; CEST = chemical exchange saturation transfer; MTR = magnetization transfer ratio asymmetry; BOLD-MRI = blood oxygen level-dependent-magnetic resonance imaging; R2* = the transverse relaxation rate; OE-MRI = oxygen-enhanced-magnetic resonance imaging; ΔT1 = changes in the longitudinal relaxation rate.

**Table 2:** The evidence strength, limitations, and clinical status of MRI techniques utilized in evaluating the tumor microenvironment in head and neck cancer.

Technique	Evidence Strength	References	Limitations	Clinical Status
DCE-MRI	Moderate to high	[8,10,11,13,14,15,16]	Pharmacokinetic model variability Field strength, temporal resolution, and contrast agent heterogeneity Non-generalizable Ktrans thresholds	Specialized centers
ASL	Low	[12,17,18,19,20,21,24]	Single versus multidelay post-labeling variability Limited head and neck specific data	Research stage
DWI/ADC	High	[26,27,28,29,30]	Variable b-value selection Non-generalizable ADC thresholds	Routine clinical use
IVIM	Moderate	[34,35,37,38,39,40,41,42]	Variable b-value number and range Non-standardized fitting algorithms Non-generalized D thresholds Timing of acquisition relative to treatment	Research stage/Specialized centers
DKI	Weak to moderate	[33,36,43,44,45]	Variable b-value protocols Acquisition timing relative to treatment	Research stage
APT/CEST	Low	[46,47,48,49,50,51]	2D versus 3D acquisition variability Magnetic field inhomogeneity	Investigational
BOLD-MRI	Very Low	[52,53,55]	R2* measurement No standardized protocols	Investigational
OE-MRI	Low	[6,52,53,54]	ΔT1 measurements No standardized protocols	Investigational

DCE-MRI = dynamic contrast enhanced-magnetic resonance imaging; ASL = arterial spin labeling; DWI/ADC = diffusion-weighted imaging/apparent diffusion coefficient; IVIM = intravoxel incoherent motion; DKI = diffusion kurtosis imaging; APT = amide proton transfer; CEST = chemical exchange saturation transfer; BOLD-MRI = blood oxygen level-dependent-magnetic resonance imaging; OE-MRI = oxygen-enhanced-magnetic resonance imaging.

## Data Availability

No new data were created or analyzed in this study. Data sharing is not applicable to this article.

## Abbreviations

The following abbreviations are used in this manuscript:

ADC	Apparent diffusion coefficient
APT	Amide proton transfer
ASL	Arterial spin labeling
AUC	Area under curve
BOLD	Blood oxygen level-dependent
CEST	Chemical exchange saturation transfer
CT	Computed tomography
D	True diffusion coefficient
D*	Pseudo-diffusion coefficient
Dapp	Apparent diffusion coefficient corrected for the non-Gaussian behavior of water molecules
DCE-MRI	Dynamic contrast-enhanced magnetic resonance imaging
DKI	Diffusion kurtosis imaging
DWI	Diffusion-weighted imaging
EBV	Epstein-Barr virus
EES	Extravascular extracellular space
f	Perfusion fraction
HPV	Human papillomavirus
IVIM	Intravoxel incoherent motion
Kapp	Apparent diffusional kurtosis parameter
Kep	Washout rate
Ki-67	Proliferation index
Ktrans	Volume transfer constant
MRI	Magnetic resonance imaging
MTRasym	Magnetization transfer asymmetry
OE-MRI	Oxygen-enhanced magnetic resonance imaging
ppm	Parts per million
r_s_	Spearman rank correlation coefficient
TBF	Tumor blood flow
TTP	Time to peak
US	Ultrasound
Ve	Extravascular extracellular volume fraction
VEGF	Vascular endothelial growth factor
ΔTBF	Changes in tumor blood flow

## References

[1] Filho A.M., Laversanne M., Ferlay J., Colombet M., Piñeros M., Znaor A., Parkin D.M., Soerjomataram I., Bray F. (2025). The GLOBOCAN 2022 cancer estimates: Data sources, methods, and a snapshot of the cancer burden worldwide. Int. J. Cancer.

[2] Sun H., Yu M., An Z., Liang F., Sun B., Liu Y., Zhang S. (2025). Global burden of head and neck cancer: Epidemiological transitions, inequities, and projections to 2050. Front. Oncol..

[3] Leemans C.R., Braakhuis B.J., Brakenhoff R.H. (2011). The molecular biology of head and neck cancer. Nat. Rev. Cancer.

[4] Mukherjee S., Fischbein N.J., Baugnon K.L., Policeni B.A., Raghavan P. (2023). Contemporary Imaging and Reporting Strategies for Head and Neck Cancer: MRI, FDG PET/MRI, NI-RADS, and Carcinoma of Unknown Primary—AJR Expert Panel Narrative Review. AJR Am. J. Roentgenol..

[5] Bonate R., Awan M., Himburg H., Wong S., Shukla M., Zenga J., Paulson E.S. (2025). Patterns of response in head and neck cancer subregions using daily Quantitative MRI from MR-guided radiation therapy. Clin. Transl. Radiat. Oncol..

[6] McCabe A., Martin S., Rowe S., Shah J., Morgan P.S., Borys D., Panek R. (2024). Oxygen-enhanced MRI assessment of tumour hypoxia in head and neck cancer is feasible and well tolerated in the clinical setting. Eur. Radiol. Exp..

[7] Garau L.M., Rensi M., Bastianutti L., Bologna R., Povolato M., Capobianco D., Gregorio F.D. (2025). The Relationship Between Functional Parameters Derived from Diffusion-Weighted MRI and 18F-Fluorodeoxyglucose PET/CT in Head and Neck Squamous Cell Carcinoma: A Systematic Review and Meta-analysis. Int. Arch. Otorhinolaryngol..

[8] Gaddikeri S., Gaddikeri R.S., Tailor T., Anzai Y. (2016). Dynamic Contrast-Enhanced MR Imaging in Head and Neck Cancer: Techniques and Clinical Applications. AJNR Am. J. Neuroradiol..

[9] Meyer H.J., Höhn A.K., Surov A. (2021). Associations between dynamic-contrast enhanced MRI and tumor infiltrating lymphocytes and tumor-stroma ratio in head and neck squamous cell cancer. Cancer Imaging.

[10] Kåstad Høiskar M., Sæther O., Delange Alsaker M., Røe Redalen K., Winter R.M. (2024). Quantitative dynamic contrast-enhanced magnetic resonance imaging in head and neck cancer: A systematic comparison of different modelling approaches. Phys. Imaging Radiat. Oncol..

[11] Longo A., Hudler P., Strojan P., Plavc G., Umek L., Popovic K.S. (2024). Predictive potential of dynamic contrast-enhanced MRI and plasma-derived angiogenic factors for response to concurrent chemoradiotherapy in human papillomavirus-negative oropharyngeal cancer. Radiol. Oncol..

[12] Tian T., Liu L., Sun J., Fu J., Luo X., Ye J., Tang G. (2026). Multidelay Pseudocontinuous Arterial Spin Labelling and Conventional Single-delay Pseudocontinuous Arterial Spin Labelling In Nasopharyngeal Carcinoma: Correlation with Dynamic Contrast-enhanced Magnetic Resonance Imaging. Semin. Ultrasound CT MRI.

[13] Smits H.J.G., Vink S.J., de Ridder M., Philippens M.E.P., Dankbaar J.W. (2024). Prognostic value of pretreatment radiological MRI variables and dynamic contrast-enhanced MRI on radiotherapy treatment outcome in laryngeal and hypopharyngeal tumors. Clin. Transl. Radiat. Oncol..

[14] Coudert H., Mirafzal S., Dissard A., Boyer L., Montoriol P.F. (2021). Multiparametric magnetic resonance imaging of parotid tumors: A systematic review. Diagn. Interv. Imaging.

[15] Espinoza S., Malinvaud D., Siauve N., Halimi P. (2013). Perfusion in ENT imaging. Diagn. Interv. Imaging.

[16] Reber B., He R., Abdelaal M.R., Mohamed A.S.R., Mulder S.L., Humbert Vidan L., Fuller C.D., Lai S.Y., Brock K.K. (2025). Post-RT Head and Neck DCE-MRI: Association Between Mandibular Dose and ve. Cancers.

[17] Iutaka T., de Freitas M.B., Omar S.S., Scortegagna F.A., Nael K., Nunes R.H., Pacheco F.T., Maia Júnior A.C.M., do Amaral L.L.F., da Rocha A.J. (2023). Arterial Spin Labeling: Techniques, Clinical Applications, and Interpretation. Radiographics.

[18] Liu J., Zhu J., Wang Y., Wang F., Yang H., Wang N., Chu Q., Yang Q. (2022). Arterial spin labeling of nasopharyngeal carcinoma shows early therapy response. Insights Imaging.

[19] Romano A., Romano A., Moltoni G., Palizzi S., Muscoli A., D’Eufemia S., Parri E., Faggiano A., Ciddio A.B., Guarnera A. (2025). Role of Pseudo-Continuous Arterial Spin Labeling and 4D MR Angiography in the Diagnosis of Neck Paragangliomas. J. Clin. Med..

[20] Nguyen H.H., Ng N.N., Awasthi S., Vogel H., Iv M. (2025). Arterial spin labeling perfusion MRI differentiates between radiation necrosis and tumor in brain metastases treated with stereotactic radiosurgery. Neuro-Oncol. Adv..

[21] Oka I., Yogi A., Ishikawa K., Heianna J., Maeda H., Nishie A. (2024). Usefulness of arterial spin labeling MR angiography as preprocedural mapping for the intra-arterial chemotherapy in patients with maxillary sinus cancer: A case report. Radiol. Case Rep..

[22] Alamolhoda F., Faeghi F., Bakhshandeh M., Ahmadi A., Sanei Taheri M., Aabbasi S. (2019). Diagnostic Value of Diffusion Weighted Magnetic Resonance Imaging in Evaluation of Metastatic Neck Lymph Nodes in Head and Neck Cancer: A Sample of Iranian Patient. Asian Pac. J. Cancer Prev..

[23] Ermongkonchai T., Khor R., Wada M., Lau E., Xing D.T., Ng S.P. (2023). A review of diffusion-weighted magnetic resonance imaging in head and neck cancer patients for treatment evaluation and prediction of radiation-induced xerostomia. Radiat. Oncol..

[24] Abdel Razek A.A.K. (2018). Arterial spin labelling and diffusion-weighted magnetic resonance imaging in differentiation of recurrent head and neck cancer from post-radiation changes. J. Laryngol. Otol..

[25] Connor S., Christoforou A., Touska P., Robinson S., Fischbein N.J., de Graaf P., Péporté A.R.J., Hirvonen J., Hadnadjev Šimonji D., Guzmán Pérez-Carrillo G.J. (2025). An international survey of diffusion and perfusion magnetic resonance imaging implementation in the head and neck. Eur. Radiol..

[26] Driessen J.P., van Kempen P.M., van der Heijden G.J., Philippens M.E., Pameijer F.A., Stegeman I., Terhaard C.H., Janssen L.M., Grolman W. (2015). Diffusion-weighted imaging in head and neck squamous cell carcinomas: A systematic review. Head Neck.

[27] Parsaei M., Sanjari Moghaddam H., Mazaheri P. (2024). The clinical utility of diffusion-weighted imaging in diagnosing and predicting treatment response of laryngeal and hypopharyngeal carcinoma: A systematic review and meta-analysis. Eur. J. Radiol..

[28] Suh C.H., Choi Y.J., Baek J.H., Lee J.H. (2018). The Diagnostic Value of Diffusion-Weighted Imaging in Differentiating Metastatic Lymph Nodes of Head and Neck Squamous Cell Carcinoma: A Systematic Review and Meta-Analysis. AJNR Am. J. Neuroradiol..

[29] Şerifoğlu İ., Oz İ.İ., Damar M., Tokgöz Ö., Yazgan Ö., Erdem Z. (2015). Diffusion-weighted imaging in the head and neck region: Usefulness of apparent diffusion coefficient values for characterization of lesions. Diagn. Interv. Radiol..

[30] Koontz N.A., Wiggins R.H. (2017). Differentiation of Benign and Malignant Head and Neck Lesions with Diffusion Tensor Imaging and DWI. AJR Am. J. Roentgenol..

[31] Chakrabarty N., Mahajan A., Agrawal A., Prabhash K., D’Cruz A.K. (2024). Comprehensive review of post-treatment imaging in head and neck cancers: From expected to unexpected and beyond. Br. J. Radiol..

[32] Fujima N., Sakashita T., Homma A., Shimizu Y., Yoshida A., Harada T., Tha K.K., Kudo K., Shirato H. (2017). Advanced diffusion models in head and neck squamous cell carcinoma patients: Goodness of fit, relationships among diffusion parameters and comparison with dynamic contrast-enhanced perfusion. Magn. Reson. Imaging.

[33] Minosse S., Marzi S., Piludu F., Vidiri A. (2017). Correlation study between DKI and conventional DWI in brain and head and neck tumors. Magn. Reson. Imaging.

[34] Xu X.Q., Choi Y.J., Sung Y.S., Yoon R.G., Jang S.W., Park J.E., Heo Y.J., Baek J.H., Lee J.H. (2016). Intravoxel Incoherent Motion MR Imaging in the Head and Neck: Correlation with Dynamic Contrast-Enhanced MR Imaging and Diffusion-Weighted Imaging. Korean J. Radiol..

[35] Fujima N., Yoshida D., Sakashita T., Homma A., Tsukahara A., Tha K.K., Kudo K., Shirato H. (2014). Intravoxel incoherent motion diffusion-weighted imaging in head and neck squamous cell carcinoma: Assessment of perfusion-related parameters compared to dynamic contrast-enhanced MRI. Magn. Reson. Imaging.

[36] Wu G., Li M.M., Chen F., Lin S.M., Yang K., Zhao Y.M., Zhu X.L., Huang W.Y., Li J.J. (2017). Diffusion-kurtosis imaging predicts early radiotherapy response in nasopharyngeal carcinoma patients. Oncotarget.

[37] Marzi S., Piludu F., Vidiri A. (2013). Assessment of diffusion parameters by intravoxel incoherent motion MRI in head and neck squamous cell carcinoma. NMR Biomed..

[38] Li Y., Liu Q., Wu W., Liu Z., Zhang Y., Dou Y., Bu Q., Zhang S. (2024). Intravoxel incoherent motion diffusion-weighted imaging (IVIM-DWI) combined with conventional MRI for the detection of skull-base invasion in nasopharyngeal carcinoma: Comparison with 18F-sodium fluoride (18F-NaF) positron emission tomography/computed tomography (PET/CT). Quant. Imaging Med. Surg..

[39] Song Q., Li F., Chen X., Wang J., Liu H., Cheng Y. (2021). Early detection treatment response for head and neck carcinomas using intravoxel incoherent motion-magnetic resonance imaging: A meta-analysis. Dentomaxillofac. Radiol..

[40] Ding Y., Hazle J.D., Mohamed A.S., Frank S.J., Hobbs B.P., Colen R.R., Gunn G.B., Wang J., Kalpathy-Cramer J., Garden A.S. (2015). Intravoxel incoherent motion imaging kinetics during chemoradiotherapy for human papillomavirus-associated squamous cell carcinoma of the oropharynx: Preliminary results from a prospective pilot study. NMR Biomed..

[41] Paudyal R., Oh J.H., Riaz N., Venigalla P., Li J., Hatzoglou V., Leeman J., Nunez D.A., Lu Y., Deasy J.O. (2017). Intravoxel incoherent motion diffusion-weighted MRI during chemoradiation therapy to characterize and monitor treatment response in human papillomavirus head and neck squamous cell carcinoma. J. Magn. Reson. Imaging.

[42] Jia Q.J., Zhang S.X., Chen W.B., Liang L., Zhou Z.G., Qiu Q.H., Liu Z.Y., Zeng Q.X., Liang C.H. (2014). Initial experience of correlating parameters of intravoxel incoherent motion and dynamic contrast-enhanced magnetic resonance imaging at 3.0 T in nasopharyngeal carcinoma. Eur. Radiol..

[43] Minosse S., Marzi S., Piludu F., Boellis A., Terrenato I., Pellini R., Covello R., Vidiri A. (2020). Diffusion kurtosis imaging in head and neck cancer: A correlation study with dynamic contrast enhanced MRI. Phys. Med..

[44] Huang N., Chen Y., She D., Xing Z., Chen T., Cao D. (2022). Diffusion kurtosis imaging and dynamic contrast-enhanced MRI for the differentiation of parotid gland tumors. Eur. Radiol..

[45] Ren W., Zheng X., Wu S., Wu C., Zheng D. (2025). Prognostic Value of Pre-Treatment Diffusion Kurtosis Imaging for Progression-Free Survival Prediction in Advanced Nasopharyngeal Carcinoma. Cancer Med..

[46] Gao T., Zou C., Li Y., Jiang Z., Tang X., Song X. (2021). A Brief History and Future Prospects of CEST MRI in Clinical Non-Brain Tumor Imaging. Int. J. Mol. Sci..

[47] Jones K.M., Pollard A.C., Pagel M.D. (2018). Clinical applications of chemical exchange saturation transfer (CEST) MRI. J. Magn. Reson. Imaging.

[48] Kamimura K., Nakajo M., Yoneyama T., Takumi K., Kumagae Y., Fukukura Y., Yoshiura T. (2019). Amide proton transfer imaging of tumors: Theory, clinical applications, pitfalls, and future directions. Jpn. J. Radiol..

[49] Yuan J., Chen S., King A.D., Zhou J., Bhatia K.S., Zhang Q., Yeung D.K., Wei J., Mok G.S., Wang Y.X. (2014). Amide proton transfer-weighted imaging of the head and neck at 3 T: A feasibility study on healthy human subjects and patients with head and neck cancer. NMR Biomed..

[50] Liu W., Wang X., Xie S., Liu W.V., Masokano I.B., Bai Y., Chen J., Zhong L., Luo Y., Zhou G. (2023). Amide proton transfer (APT) and magnetization transfer (MT) in predicting short-term therapeutic outcome in nasopharyngeal carcinoma after chemoradiotherapy: A feasibility study of three-dimensional chemical exchange saturation transfer (CEST) MRI. Cancer Imaging.

[51] Liu X., Li P., Shang P., Lu J., Ai K., Ma X. (2026). Magnetic resonance imaging differentiating benign from malignant bone and soft tissue tumors and assessing Ki-67 expression using APT and DWI tools. Front. Oncol..

[52] Huang W., Li N., Zhu S., Zhang Y., Song X., Zhang B., Yan H., Tian J., Wang K., Zhang S. (2025). Imaging hypoxia for head and neck cancer: Current status, challenges, and prospects. Theranostics.

[53] O’Connor J.P.B., Robinson S.P., Waterton J.C. (2019). Imaging tumour hypoxia with oxygen-enhanced MRI and BOLD MRI. Br. J. Radiol..

[54] Dubec M.J., Price J., Berks M., Gaffney J., Little R.A., Porta N., Sridharan N., Datta A., McHugh D.J., Hague C.J. (2024). Oxygen-Enhanced MRI Detects Incidence, Onset, and Heterogeneity of Radiation-Induced Hypoxia Modification in HPV-Associated Oropharyngeal Cancer. Clin. Cancer Res..

[55] Sidhu H.S., Price D., Beale T., Morley S., Adeleke S., Papoutsaki M.V., Forster M., Carnell D., Mendes R., Taylor S.A. (2025). Oxygen-Enhanced R2* Weighted MRI and Diffusion Weighted MRI of Head and Neck Squamous Cell Cancer Lymph Nodes in Prediction of 2-Year Outcome Following Chemoradiotherapy. Cancers.

[56] Shukla-Dave A., Obuchowski N.A., Chenevert T.L., Jambawalikar S., Schwartz L.H., Malyarenko D., Huang W., Noworolski S.M., Young R.J., Shiroishi M.S. (2019). Quantitative imaging biomarkers alliance (QIBA) recommendations for improved precision of DWI and DCE-MRI derived biomarkers in multicenter oncology trials. J. Magn. Reson. Imaging.

[57] Marzi S., Piludu F., Forina C., Sanguineti G., Covello R., Spriano G., Vidiri A. (2017). Correlation study between intravoxel incoherent motion MRI and dynamic contrast-enhanced MRI in head and neck squamous cell carcinoma: Evaluation in primary tumors and metastatic nodes. Magn. Reson. Imaging.

[58] Guo W., Zhang Y., Luo D., Yuan H. (2020). Dynamic contrast-enhanced magnetic resonance imaging (DCE-MRI) for pretreatment prediction of neoadjuvant chemotherapy response in locally advanced hypopharyngeal cancer. Br. J. Radiol..

[59] Paolisso P., Foà A., Bergamaschi L., Graziosi M., Rinaldi A., Magnani I., Angeli F., Stefanizzi A., Armillotta M., Sansonetti A. (2023). Echocardiographic Markers in the Diagnosis of Cardiac Masses. J. Am. Soc. Echocardiogr..

